# How Cancer Survivors Provide Support on Cancer-Related Internet Mailing Lists

**DOI:** 10.2196/jmir.9.2.e12

**Published:** 2007-05-14

**Authors:** Andrea Meier, Elizabeth J Lyons, Gilles Frydman, Michael Forlenza, Barbara K Rimer

**Affiliations:** ^4^Health Sciences DepartmentSimon Fraser UniversityVancouverBCCanada; ^3^Association of Cancer Online Resources (ACOR)New YorkNYUSA; ^2^School of Public HealthDepartment of Health Behavior and Health EducationThe University of North CarolinaChapel HillNCUSA; ^1^School of Social WorkThe University of North CarolinaChapel HillNCUSA

**Keywords:** Internet, cancer, patients, survivors, online communities, mailing lists, online support groups, qualitative research

## Abstract

**Background:**

Internet mailing lists are an important and increasingly common way for cancer survivors to find information and support. Most studies of these mailing lists have investigated lists dedicated to one type of cancer, most often breast cancer. Little is known about whether the lessons learned from experiences with breast cancer lists apply to other cancers.

**Objectives:**

The aim of the study was to compare the structural characteristics of 10 Internet cancer-related mailing lists and identify the processes by which cancer survivors provide support.

**Methods:**

We studied a systematic 9% sample of email messages sent over five months to 10 cancer mailing lists hosted by the Association of Cancer Online Resources (ACOR). Content analyses were used to compare the structural characteristics of the lists, including participation rates and members’ identities as survivors or caregivers. We used thematic analyses to examine the types of support that list members provided through their message texts.

**Results:**

Content analyses showed that characteristics of list members and subscriber participation rates varied across the lists. Thematic analyses revealed very little “off topic” discussion. Feedback from listowners indicated that they actively modeled appropriate communication on their lists and worked to keep discussions civil and focused. In all lists, members offered support much more frequently than they requested it; survivors were somewhat more likely than caregivers to offer rather than to ask for support. The most common topics in survivors’ messages were about treatment information and how to communicate with health care providers. Although expressions of emotional support were less common than informational support, they appeared in all lists. Many messages that contained narratives of illness or treatment did not specifically ask for help but provided emotional support by reassuring listmates that they were not alone in their struggles with cancer. Survivors’ explicit expressions of emotional support tended to be messages that encouraged active coping. Such messages also provided senders with opportunities to assume personally empowering “helper” roles that supported self-esteem.

**Conclusions:**

Many cancer survivors use the Internet to seek informational and emotional support. Across 10 lists for different cancers, informational support was the main communication style. Our finding of an emphasis on informational support is in contrast to most prior literature, which has focused on emotional support. We found the most common expressions of support were offers of technical information and explicit advice about how to communicate with health care providers. Topics and proportions of informational and emotional support differed across the lists. Our previous surveys of ACOR subscribers showed that they join the lists primarily to seek information; this qualitative study shows that they can and do find what they seek. They also find opportunities to play rewarding roles as support givers.

## Introduction

The importance of the Internet as a health resource is demonstrated by the fact that 8 out of 10 Internet users in 2005 reported looking for health information online, most commonly seeking information on specific diseases and certain medical treatments [1]. In the United States, it is estimated that 56.3 million people actively seek information about chronic diseases [2], and the information they gather affects their health choices [3, 4]. Internet users report employing the information they find on the Internet to diagnose health problems, enhance their medical care, and to validate the advice they receive from doctors [5].

An estimated 100 million Americans report ever having been members of some type of online group, and 79 million Americans have become members of online support groups [6]. On the popular Yahoo site alone, users can choose from over 30000 health-related support groups. The World Wide Web and email now permit a variety of group communication formats, many of which are widely used and have been described in detail elsewhere [7]. Here, we will describe our work with one type of group format—the mailing list, also known as an email discussion group. In Internet mailing lists, email messages (asynchronous communication) from authorized senders (subscribers) may convey information and support to other list subscribers. In the case of eHealth support lists, many subscribers are living with similar health conditions or are caregivers to survivors.

Some sources estimate that as many as 1 in 4 disease information seekers join online discussion groups [8]. Approximately 23 million people are reported as very active in online communities [9]. Our recent count of 33000 health-related online self-help groups on Yahoo shows that participation in electronic support groups (ESGs) continues to grow. This estimate represents a 32% increase over the number reported by Eysenbach and colleagues in 2004 [10]. Although estimates vary greatly, millions of people in the United States and, increasingly, around the world are turning to online support groups to deal with health concerns. (Most online support lists are hosted in the United States but are accessible outside US boundaries. For a description of online support groups sited outside the United States, see [11]).

### ESGs Within Virtual Social Networks

There is an ongoing debate about whether support lists should be considered ESGs, informal grass roots virtual organizations, or electronically networked communities.

#### Mailing Lists as Support Groups

Support mailing lists are similar to traditional offline self-help groups in that they are “composed of members who share a common condition, situation, heritage, symptom or experience [12].” eHealth support lists and offline self-help groups share the goal of helping people learn about and cope with a variety of risk factors, diseases, and conditions.

Typically, offline face-to-face support groups are small, composed of 10 to 12 members. In face-to-face groups, the small size makes it easier for members to interact with each other, to build trusting relationships, and for the groups to become cohesive [13]. By contrast, online mailing lists can have hundreds or even thousands of members, many of whom post messages infrequently, if ever. Using a liberal definition of participation—at least one post within a three-month period—Nonnecke and Preece found that only about 55% of subscribers to a virtual health support group could be described as active participants [14]. Those members who post with some regularity often become acquainted and emotionally bonded with each other, forming subgroups that function like cohesive face-to-face support groups. The impact of participation on lurkers, those who read messages but don’t write them, is unknown.

Currently, most ESGs appear not to be professionally facilitated but rely on peer leaders, making them more like self-help or mutual aid groups than professionally facilitated face-to-face support group interventions. The Association of Cancer Online Resources (ACOR) mailing lists we studied follow the peer leader model. Many of the listowners are extremely knowledgeable about health and cancer. These peer-leader listowners intervene both online and offline as needed to correct misconceptions, enforce group norms, and provide information, but they aim to do so as infrequently as possible [12].

#### Support Lists as Grassroots Organizations and Virtual Communities

The list subgroups described above exist concurrently within larger networks that resemble grassroots organizations. Because mailing lists are embedded in the Web, members can follow links to additional information sources. In many ways, lists act as a portal for members, leading them to further explore the Web and to discover the external, socioeconomic, and structural factors that contribute to their health concerns. In this respect, support lists are like their offline grassroots counterparts in that they can organize around “communities of interest” to address social injustice. (In this instance, the injustice involves the unmet needs for support, access to treatment, and resources for cancer-affected people [15, 16]). As in offline grassroots organizations, support lists tend to be composed of members who share similar concerns, and the groups’ informal organizational structures enable quick response to changing circumstances.

### Research on Therapeutic Factors and Outcomes of ESG Participation

Anecdotal and descriptive information about online self-help processes suggest that virtual communities are possibly the most important aspect of the Web, with the biggest impact on health outcomes [10]. Research in this area is still in its early development; consequently, rigorous studies documenting these benefits are difficult to find. Much of the research to date has focused on describing how social support is communicated online [17,18] or how Internet communication has made it possible to offer support to greater numbers of people—especially those with rare diseases [19,20]—in ways that are satisfying and empowering to most participants [18,21-23]. Because ESG participants are invisible to each other, it is easier for members to communicate about common concerns. Participants, particularly patients in illness support groups, do not have to be concerned whether their personal appearance will affect others’ reactions to them, and race, gender, and other sociodemographic differences are not immediately apparent [24]. Members may increase their self-confidence by becoming better informed about their illnesses. These processes appear to enhance self-esteem and increase participants’ comfort level in dealing with health professionals [5]. Participation in ESGs may help cancer survivors find information, obtain support, formulate questions to ask health care providers, and become more active partners in their care decisions [25]. However, while prior reports are encouraging regarding the impact of ESGs, the data were from uncontrolled studies.

### Research Methodology and ESGs

Researchers have found naturally forming online groups that offer peer-to-peer social support difficult to study using conventional methods because both format and content are difficult to replicate using controlled methods [10]. Some have tried to cope with this methodological challenge by forming project-specific ESGs as components of multi-modality intervention studies. Typically, these ESGs have used closed memberships, trained facilitators, and limited brief durations. In this respect, such project-specific ESGs used as formal interventions have greater similarity to face-to-face support groups [13].

In a recent systematic review of 38 studies on the effects of peer-to-peer interactions in health-related virtual communities and ESGs, Eysenbach and colleagues concluded that only six studies evaluated pure peer-to-peer communities [10]. In the Eysenbach review, qualifying studies tended to have “less than optimum research designs” in that they were exploratory in nature, used nonexperimental designs, and had small sample sizes. One study had a 2 × 2 factorial design (full version website or control group website combined with or without peer-to-peer groups) that allowed an evaluation of the peer-to-peer component; the 31 remaining studies evaluated complex interventions in which online communities were only an adjunct to broader interventions [10]. These findings were similar to those of an earlier systematic review of the research on online cancer support groups (published from 1993-2002) conducted by Klemm and colleagues [17].

In the six peer-to-peer community studies included in the Eysenbach review, the type of ESGs varied across studies and included Web-based discussion forums, chat groups, combinations of chat and newsgroups, mailing lists, and one voice bulletin board system. All ESGs included in these studies had some degree of formal facilitation by health professionals. The role of health professionals as facilitators was to stimulate discussions by posing questions to the group, to post topics of interest to the group, or to provide educational materials. Some studies that used project-specific ESGs observed a possible dose-response effect between higher rates of participation and better outcomes for problems such as depression [26,27], caregiver strain [28], and increased perceived social support among people with diabetes [29]; however, the direction of causality is uncertain. Given the dearth of research in this field, we can only affirm that findings about the benefits of ESGs are promising but inconclusive. It is worth mentioning that Eysenbach and colleagues noted that no negative effects were reported. They further concluded that, because of the complexity of ESGs and methodological challenges, lack of evidence should not lead to the conclusion that ESGs are ineffective [10]. Rather, there is insufficient evidence regarding their efficacy. These cumulative findings suggest that ESGs—both pure peer-to-peer and short-term interventions—may play important mediating and moderating roles, creating the conditions that promote participant self-efficacy and positive health behaviors [10,29]. This research focuses on the extent to which peer-to-peer groups for different types of cancer contribute to these constructive attitudes and behaviors.

### Conceptual Framework

**Figure 1:**
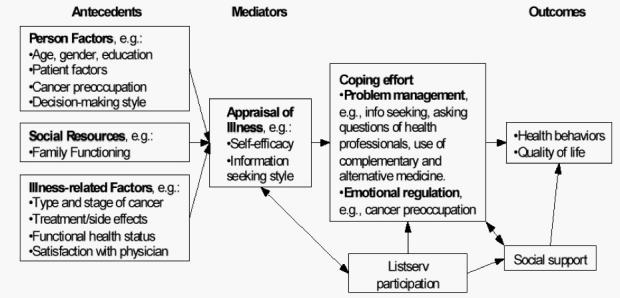
Simplified model of stress and coping (Adapted from stress and coping models developed by Northouse LL, Caffey M, Deichelbohrer L, et al. The quality of life of African American women with breast cancer. Research in Nursing and Health 1999;22:449-460 and Wenzel L, Glanz K, Lerman C. Stress, coping and health behavior. In K Glanz, BK Rimer, FM Lewis, editors. Health Behavior and Health Education. San Francisco, CA: Jossey-Bass; 2003:210-239)

The underlying conceptual framework for the Health eCommunities Project (for detailed description see [30]) was applied in developing the initial coding framework for our analysis. Briefly, the framework is informed by a general model of stress and coping [31-33] based on Lazarus and Folkman’s theory of stress and coping [34] (Figure 1). Coping is a process of managing stress, initiated when a person appraises problems as exceeding their individual resources [35]. Here, we use two major categories of coping: problem-focused and emotion-focused [34]. Problem-focused coping entails constructive action to change the stressful situation. In the context of online mailing lists, problem-focused coping might be seeking treatment information online or researching complementary and alternative medicine. Emotion-focused coping uses actions to change an individual’s emotional response to the stressful situation. Emotion-focused coping in an online community might occur when a member vents strong emotions by writing about them or when he or she avoids or denies such feelings.

Either coping strategy can lead to positive or negative outcomes depending on how well matched it is to the situation. According to this framework, when people confront stress, personality characteristics, external resources, and social support can influence coping, thereby mediating the effect on psychological outcome [36]. Social support can facilitate an individual’s positive efforts to cope, potentially bolstering both positive problem-focused and emotion-focused coping.

### The Health eCommunities Study

The Health eCommunities study is a multi-method project, using both quantitative and qualitative methods, designed to assess the impact on cancer survivors and their caregivers of participating in mailing lists sponsored by ACOR. Health eCommunities represents a collaboration between the ACOR leadership and an interdisciplinary team from the University of North Carolina at Chapel Hill (UNC). An overview of the study and preliminary survey findings have been reported elsewhere [30].

Established in 1996, ACOR is a non-profit Web portal that offers users access to a rich array of information and support for cancer survivors, family members, friends, health care professionals, and researchers. The website also provides links to information about treatment options, clinical trials, and cancer-related books. As an organization, ACOR is a loose confederation of more than 150 publicly accessible mailing lists that range in size from very small (< 10 subscribers) to very large (> 2000 subscribers). The mailing lists are run by dedicated volunteer teams of listowners, most of whom are cancer survivors or surviving spouses or friends of deceased ACOR members. ACOR capitalizes on listowners’ expertise by offering listowners their own mailing list in which to discuss shared problems in list management and facilitation. In addition to running their own lists, listowners may serve on one or more other ACOR listowner teams.

This paper focuses on the qualitative component of the study, including content and thematic analyses of email message texts exchanged by ACOR members (survivors and caregivers) in 10 lists. Recent technological innovations in automatic text analysis now make large-scale studies of support lists possible [37]. Our project is unique among the body of qualitative studies of mailing list–based email correspondence (most using manual coding) because it represents one of the largest samples of message texts of ESGs for related illnesses (eg, different types of cancer).

Because relatively little is known about ESG functioning, we began with two global questions: (1) What are the major concerns of ACOR members? and (2) What kinds of support do these groups offer? However, an additional focus of this paper reflects our unexpected finding that list members were more likely to send messages offering support than they were to request support. This finding was unexpected because previous studies of ESGs reported that most Internet users go online seeking both information and support; therefore, we expected that ACOR members would request support at least as often as they offered support. Further, list members who had the most direct need for support, the cancer survivors, were much more likely to offer support more often than listmates who were caregivers. For these reasons, this paper is devoted to an examination of cancer survivors’ characteristics across the 10 ACOR lists, their patterns of supportive behavior, and the ways they used their collective Internet connections to promote their own and others’ ability to cope.

## Methods

### Data Collection

#### Selection of Participating Mailing Lists

Although ACOR permitted our research team access to ACOR as an online health community, individual listowners made the ultimate decision whether their respective lists would participate. We used three criteria to select ACOR lists. First, they had to have listowners with sufficient experience and confidence in their roles as listowners to be good research collaborators. Using his knowledge of the listowners, Frydman identified likely volunteers, encouraged their participation, and worked with the UNC team to gain listowners’ consent. Second, lists were selected to represent a range of cancer diagnoses and likely prognoses. Finally, because the project’s survey component would focus on new members, lists were selected based on their history of accruing new members. Although we knew that caregivers were active participants on these lists, we limited heterogeneity of the sample at the group level by excluding lists organized exclusively for cancer caregivers.

#### Sampling List Messages

For the qualitative study, we used individual email messages as the units of analysis. We sought to obtain enough messages to tap into the diversity of members’ views, to capture differences in message content between very active and less active list participants, and to allow for stratification of survivor and caregiver data. Ultimately, we drew our data from the set of messages sent to the 10 lists over a five-month period between November 2003 and March 2004.

Mailing lists appear to have a life cycle of growth and decline, with the number of active members declining as the number of subscribers increases [38]. We did not have full baseline data on subscriber activity levels for the study lists in the months before the study began. One member of the team had been given permission to access the colon cancer archives for a previous study. We conducted pilot analyses of the colon cancer list participation data from November 2002 to March 2003 to estimate how many messages would be in the overall sample. Based on colon list data, we used a conservative estimate of one message per month per subscriber to define active membership.

Currently, there is no consensus among researchers about appropriate strategies for sampling the behavior of list participants. We chose to systematically sample messages sorted chronologically, assuming this procedure would enable us to tap messages from most active members and dominant discussion threads. Based on our pilot studies of the colon cancer list participation rates, we estimated that approximately 25% of subscribers would send a minimum of one message per month. Based on 9881 subscribers in the 10 lists in November 2003, we estimated that over 12000 messages would be sent during the five-month data collection period. Limited resources precluded analyzing all these messages; thus, we systematically sampled 9% of all archived messages for each group, anticipating a sample of 1112 messages.

ACOR staff used a multi-stage procedure to extract messages from the portal’s archives. After sorting each month’s messages chronologically, they used an automated system to extract every eleventh message sent by members (listowner messages were extracted in a separate step). Each month’s message texts were compiled into a single email digest message and emailed to the UNC research team for review and analysis. Our data collection strategy was approved by the Institutional Review Board of the UNC School of Public Health.

#### Protecting Participants’ Rights

By sampling messages from the ACOR archives instead of ongoing discussions on ACOR lists, we tried to avoid the possibility that participants would be deterred from posting to their respective lists, inadvertently disrupting their access to on-list support [39]

All listowners of the participating lists gave their permission to access their lists’ archives. Prior to the start of the Health eCommunities project, they posted messages to their lists notifying subscribers about the project and directing them to the project’s Web page for Frequently Asked Questions. Members were reminded that ACOR archives were accessible to the public.

Members were also told how sender anonymity would be protected. All message texts were de-identified by ACOR staff before the monthly message digests were transmitted to the research team. An automated process assigned each ACOR subscriber a unique ID number and removed subscribers’ names and email addresses from message headers, message texts, and signature lines sent to the UNC team. Although we only coded original messages, for message texts that were replies to other messages, we retained the original message texts in the data set so we could correctly interpret the replies. Listmembers were offered an “opt out option.” Those who wanted to have their messages removed from the data set could notify ACOR. In turn, ACOR staff would notify the UNC team about the list and participant ID numbers that would be affected.

### Analytic Approach

As in most email communication, message texts in ACOR lists were informal and loosely organized [37], making analyses challenging. Messages also varied in length. Thematically, they were pastiches that could contain technical information about treatments, side effects, clinical trials, empathic comments, requests for information, or meta-comments about group processes.

#### Content Analyses

We conducted content analyses on each message to determine the relationship of the message sender to the cancer survivor (eg, survivor, caregiver, trusted other, or health care provider) and how often each sender corresponded with his or her respective list. A significant proportion of messages lacked sufficient information to deduce the sender’s role. Subsequently, we reduced the number of messages with unknown senders by compiling all messages with the same sender ID numbers. In many cases, we could find one or more messages within those compiled subsets that contained enough information to recode all messages with identical ID numbers. During this process, we also discovered a number of cases where caregivers and survivors were sending from the same email address, as occurs when multiple family members share a common email address.

Frequencies of topics we report may not represent equivalent amounts of text. The unit of analysis was an entire message. We used Atlas.ti [40], a qualitative data analysis program, that allowed us to code each message text in many different ways. Thus, in a given message, a code could apply to a single sentence or many sentences depending on how extensively the sender addressed a topic.

For thematic analyses, we used both theory-based [41] and grounded theory [42] approaches. For theory-based analyses, we initially developed global codes based on the sensitizing concepts of the project’s simplified Stress and Coping Model (see Figure 1). Definitions for these coding categories were developed from items used in the project’s online surveys. Following principles of grounded theory [43], we immersed ourselves in the texts, refined our code definitions, and developed new codes as new themes emerged. In general, global codes developed from the model captured the major domains of message content. However, over time, it became clear that we needed subcodes to categorize emerging components of the most frequently used codes.

We searched for overarching themes of support that reflected concerns of most groups. To be selected as an overarching theme on survivor supportive communication, a given topic had to be discussed in at least three messages in a majority of the 10 lists. To be included as a subtheme, a topic had to be discussed in at least three groups.

#### Maintaining Analytical Rigor

After testing global codes on pilot data, email digests from the lists were assigned to one of two coders (AM and EJL); each coder analyzed data from five of the lists. Following procedures of constant comparison, they met regularly to discuss and resolve coding issues. To determine consistency of codes across mailing lists, we conducted tests of interrater reliability on a 10% sample of each list. Reliability was assessed using a variant of the code checking strategy recommended by Miles and Huberman [44]. For each code, the percentage of agreement was calculated using as the baseline all the instances of text where at least one rater applied that code.

## Results

### Interrater Reliability

Miles and Huberman [44] recommend minimum interrater reliability levels of .70 for each code. Using this standard, our reliability levels were in the acceptable range. For the eight major codes, average reliability was .84. For the 44 subcodes, reliability was only slightly lower at .83.

### Sample Characteristics

Sample size in online mailing list communication can be characterized along two dimensions: number of messages posted and the unduplicated number of message senders.

#### Number of Messages

**Figure 2:**
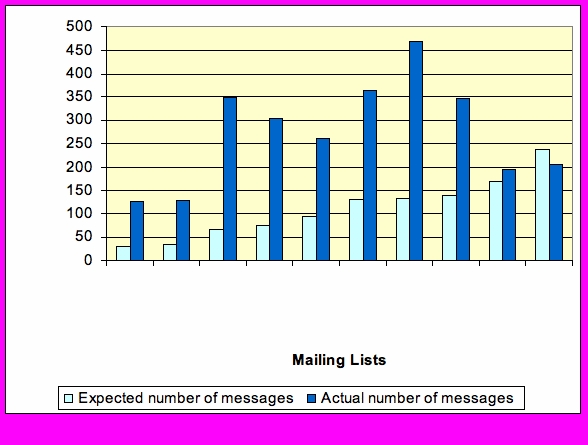
Participant activity levels: expected and actual number of messages

Participants in the sample sent nearly 50% more messages (n = 2755) than our pilot estimates of 1112 messages (Figure 2). However, these overages were not evenly distributed across lists. The ovarian cancer list sample was more than 50% larger than expected (157%), and the leiomyosarcoma (LM sarcoma) list sample was over four times larger (427%) than expected; the chronic lymphocytic leukemia (CLL) list was the only one in which the number of messages was slightly lower than expected (−13%). There was no statistical relationship between average rates of participation and five-year survival rates as defined by Surveillance, Epidemiology, and End Results (SEER) data [45].

**Figure 2.** Participant activity levels: expected and actual number of messages

#### Number of Unduplicated Senders

Although subscribers can benefit from reading messages but not sending them (also known as “lurking”), in this study, we defined participation in terms of posted messages. When we started collecting data, the 10 lists had 10153 subscribers (Table 1). The number of individual senders varied by list from 63 (non-small cell lung cancer [lung NSCLC] and long-term survivors [L-T survivors]) to 164 (ovarian cancer). Smaller lists tended to have a greater proportion of active members.

**Table 1:** Comparison of mailing list size and number of senders in sample

**Mailing List**	**Number of Members at Start-Up**	**Number of Senders in Sample (%)**
L-T survivors	277	63 (23)
Lung NSCLC	310	63 (20)
LM sarcoma	587	95 (16)
Colon	670	115 (17)
Kidney	846	137 (16)
Esophageal	1160	127 (11)
Myeloma	1191	118 (10)
Ovarian	1503	164 (11)
Prostate	1503	108 (7)
Chronic lymphocytic leukemia (CLL)	2106	135 (6)

### Participant Characteristics

Table 2 summarizes ACOR list participants’ characteristics in terms of their relationship to cancer survivors. There were statistically significant differences in the composition of the lists in terms of the participation rates of types of participants (*χ* ^2^_18_ = 389.6; N = 2967; *P* <.001), with survivors posting more often than other types of list members. In the typical group, messages from identifiable survivors made up slightly more than half (median 57%) of all correspondence, ranging from 38% (esophageal cancer) to 84% (ovarian cancer). Messages from identifiable caregivers made up slightly more than a quarter of the correspondence (median 27%), ranging from 5% (ovarian) to 48% (esophageal). Messages from other identifiable types of participants (friends and health care providers) were rare. Messages from participants who could not be identified typically made up slightly more than 13% (median) of the correspondence; this ranged from 8% (lung NSCLC) to 20% (CLL) of all messages sent.

**Table 2:** Mailing list characteristics: participation rates of different sender types and exchange of support

	Mailing List^*^										***χ*^2^ (df) ^†^**	***P***
	**CLL**	**Colon**	**Esophageal**	**Kidney**	**LM Sarcoma**	**L-T Survivors**	**Lung NSCLC**	**Myeloma**	**Ovarian**	**Prostate**		
**Participation Rates**											389.6 (18)	<.001
Patient/survivor	126 (61)	152 (49)	140 (38)	124 (47)	229 (66)	99 (75)	60 (45)	248 (53)	449 (84)	126 (63)		
Caregiver	32 (16)	120 (39)	176 (48)	92 (35)	66 (19)	17 (13)	60 (45)	163 (35)	25 (5)	28 (14)		
Other	47 (23)	38 (12)	54 (14)	46 (18)	54 (15)	16 (12)	13 (10)	59 (12)	62 (11)	46 (23)		
**Overall Support**											66.17 (9)	<.001
Explicit requests	35 (39)	17 (17)	70 (50)	22 (25)	39 (28)	6 (12)	11 (25)	37 (24)	40 (18)	21 (26)		
Explicit offers	54 (61)	84 (83)	71 (50)	67 (75)	98 (72)	45 (88)	33 (75)	119 (76)	185 (82)	59 (74)		
**Type of Support**											33.14 (9)	<.001
Emotional	24 (26)	23 (22)	25 (30)	18 (19)	45 (30)	15 (29)	5 (11)	22 (13)	63 (29)	9 (10)		
Informational	69 (74)	82 (78)	58 (70)	78 (81)	107 (70)	37 (71)	40 (89)	141 (87)	153 (71)	78 (90)		
**Emotional Support**											8.88 × 10^-8^	.105
Explicit offers	19 (79)	22 (96)	24 (96)	17 (94)	35 (78)	15 (100)	4 (80)	20 (91)	58 (92)	9 (100)		
Explicit requests	5 (21)	1 (4)	1 (4)	1 (6)	10 (22)	0 (0)	1 (20)	2 (9)	5 (8)	0 (0)		
**Informational Support**											30.10 (9)	<.001
Explicit offers	38 (55)	65 (79)	53 (91)	56 (72)	71 (66)	31 (84)	29 (73)	102 (72)	120 (78)	56 (72)		
Explicit requests	31 (45)	17 (21)	5 (9)	22 (28)	36 (34)	6 (16)	11 (27)	39 (28)	33 (22)	22 (28)		

*Numbers are reported as raw counts with percentages in parentheses. Percentages are of total messages for participation rates, total messages coded as containing support for overall support, and total messages containing emotional support and informational support for those two categories.

^†^Chi square values are Cochran-Mantel-Haenszel general association statistics with degrees of freedom in parentheses, except for Emotional Support, which uses Fisher exact test and reports table probability rather than a chi square value.

### Mailing Lists as Sources of Social Support

Table 2 further shows the absolute numbers and percentages of messages containing explicit requests for and offers of support across all lists. We also found a substantial number of instances in which participants posted messages that could be construed as *implicitly* offering support although they were not specifically responses to others messages (range: lung NSCLC 28% to ovarian 53%). Such messages included descriptions of cancer-related events without comment or requests for help. Some senders wrote narratives about their diagnosis or staging, their experiences with different treatments, and treatment side effects. Other members posted URLs to link members with information about cancer treatments, clinical trials, or new research findings.

Some messages used automated message signature files that contained thumbnail histories of survivorship experiences of senders or survivor relatives (Textbox 1). Both survivors and caregivers used this signature line feature, and they included updateable, telegraphic lists of senders’ (or their relatives’) date of diagnosis, cancer treatments and outcomes, and current health status. Although some other support lists require members to include case history information in their signature lines as bona fides of members of their lists [A Jones, personal communication, March 1, 2005], ACOR lists do not require this. Of 1751 messages sent by identifiable survivors, 267 (16%) had case history signature lines.

Previous research on asynchronous Internet-mediated communication found that correspondents rapidly develop an idealized sense of intimacy [46]. We found the intimacy in our sample of ACOR lists tended to be focused on the pragmatic details of life with cancer. Furthermore, we found surprisingly few disclosures of events in members’ lives that were unrelated to cancer survivorship.

**Four examples of survivors’ signature line texts (in order, from briefest to more detailed)**

**Table:** 

No.	**Sample Signature Line Text**
1	Rectal Duke B2; Cancerteer [sic] since 2000
2	[city, state]. (A 3-1/2 yr Fighting Four)
3	dx [date] from a Pap test, TAH/BSO, papillary serous adenocarcinoma, primary peritoneal, IIIa grade 3, 7 rounds taxol/carbo. Recurrence #I dx 9/99 from a Pap test, TAH/BSO, papillary serous adenocarcinoma, primary peritoneal, IIIa grade 3, 7 rounds taxol/carbo. Recurrence #I [date], 8 taxol/carbo. Recurrence #II [date] biopsy says estrogen + tamoxifen, 8 taxol/carbo.
4	[date] - X-rays showed spot on left lung [date] - Surgery tumor & lower lobe-left lung removed; not clear margins; 5-6 nodules like grapes 15 x 10.5 x 8.0 cm mitotic rate was 4-5/50 hpf [date] - Cat scan chest & abdomen showed tumor in or near left lung [date] - Cat scan of Head and Pelvis clear [date] - Surgery 1 tumor removed from lining of ribs; not clear margins; surgeon recommends radiology; Size: 2.8 cm x 2.2 cm x 1 cm; mitotic rate. increased to 2-3/10 hpf; & more cellular specimen. [date] - Sarcoma Clinic dx: Smooth muscle tumor of uncertain malignant potential/grade 1 leiomyosarcoma (from oncologist report to my surgeon & GP). No stage reported: when pressed Medical Onc. Said maybe 2 or 3. [date] - No adjuvant therapy; 6 mths NED; Rad Onc says Low Grade LMS needs close follow up. My surgeon will attend to follow up Scans and Xrays from now on. [date] - xray in Sept clear 9 months NED [date] - CatScan to be done in Dec/03

### Information and Advice Themes

Because our surveys of new subscribers found that cancer survivors join ACOR lists mainly because they are looking for information , it was not surprising that this theme was dominant across lists in our sample (see Table 2). The most common kind of support offered by survivors was information and advice based on their experience. We identified four major themes associated with survivors’ offers of information and advice: (1) specific treatments, (2) communicating with health care providers to find the best treatment, (3) problem management strategies, and (4) coping with cancer recurrence. Each of these themes was subdivided into several related components. Table 3 lists the major themes, associated subthemes, and the total number of lists in which survivors sent supportive messages addressing each theme.

Our sample of messages included relatively little discussion about the experience of being diagnosed. Although survivors’ messages with case history signature lines often included the diagnosis date, slightly less than 10% of messages (79 of 811) from survivors mentioned their diagnosis experience.

**Table 3:** Information and advice themes

**Theme Number**	Themes and Subthemes^*^	**Number** **of Lists** ^†^
**1.1**	**Specific treatments**	**10**
	Case histories	10
	Treatment types	9
	Factors to consider in making treatment decisions	9
**1.2**	**Communicating with health care providers to obtain good care**	**9**
	Communication factors that could affect quality of care	7
	Strategies for obtaining good cancer care	5
**1.3**	**Problem management strategies**	**8**
	Using the Internet for due diligence	8
	Using the Internet to get good cancer care and social support	4
**1.4**	**Coping with cancer recurrence**	**6**
	Coping with risk of recurrence	5
	Living with relapses	3

^*^To be selected as a major theme (in bold), a given topic had to be discussed in at least three messages in a majority of the 10 lists. To be included as a subtheme, a topic had to be discussed in at least three groups.

^†^Numbers of discussion subthemes in lists do not necessarily equal the total number of mailing lists in the major theme. These counts represent the union of the set of *all* mailing lists in which messages addressed some aspect of the major theme, while subtheme occurrences may only have been found in a subset of the mailing lists.

#### Theme 1.1: Specific Treatments

Most new subscribers reported that one of their main reasons for joining ACOR lists was to seek information and guidance about specific treatments [30]. Theme 1.1, Specific Treatments, dominated discussion in all 10 lists. There were three subthemes: case histories, information about types of treatment, and factors to consider when making treatment decisions.

In all lists, members often discussed their case histories. These messages contained accounts rich in information that other members could use to estimate the likelihood of long-term survival. In nine lists, survivors offered information about treatments and factors to consider when making treatment decisions. In discussions of treatment types, senders provided information to help listmates understand the bewildering array of options; of these, chemotherapy was mentioned most frequently. In about a third of the messages, members discussed two or more kinds of treatment options and frequently discussed how they were used in combination.

Survivors’ messages often offered help to listmates in thinking through the factors to consider when making treatment decisions and typically urged the newly diagnosed to practice “due diligence” by becoming informed consumers of cancer care. Due diligence included seeking information on treatment response rates, probability of survival or cure, likelihood of recurrences, long-term survival rates, and cancer stages. To be informed consumers, newly diagnosed members were advised to seek information on the types and severity of side effects and after effects of treatment and the likelihood of cure associated with the specific treatments being proposed and relative to other available treatment options. In some cases, senders provided information they thought listmates needed, describing their experiences with treatments, side effects, and strategies for minimizing discomfort. Senders also provided assistance for those further along in their disease progression and treatment cycles. Here, discussions addressed such diverse issues as how risk of recurrence is assessed, how to use test results to decide on whether to use standard or aggressive treatments, potential benefits of adjuvant treatments, and sequencing of treatments for different stages of cancer (eg, reserving certain chemotherapy regimens in the event the cancer became inoperable).

#### Theme 1.2: Communicating With Health Care Providers to Obtain Good Care

In nine lists, survivors offered information and advice on issues related to the role of good communication in obtaining optimal cancer care. This theme included two subthemes: the importance of communicating with health care providers and recommendations about how to obtain good cancer care.

In seven lists, survivors discussed communication factors that could affect quality of care. They advised listmates to take initiative and raise issues with their doctors to understand the relationship between disease progression and treatment options, to be prepared for treatment side effects, and to understand how to maximize quality of life. Senders provided examples to distinguish between good and bad communication with health care providers, and they described how their relationship with their provider had benefited through good communication.

Discussions about problematic communications with health care providers occurred in half the lists. Members shared examples of poor communication and bad advice received from health professionals. They offered suggestions for critically assessing the information and advice they received and encouraged listmates to use lists to clarify and validate professional opinions. Some senders posted messages expressing concern about the information or recommendations other members reported from their doctors, urging them to seek second opinions. They also offered suggestions for coping with adverse consequences of inappropriate treatment decisions.

In five lists, members offered advice about strategies to obtain good cancer care and identified treatment centers and doctors who provided good treatment. Senders offered advice on building a good medical team, how to work with team members, and how to cope with the complexity of information from different team members.

#### Theme 1.3: Problem Management Strategies

Survivors in eight lists shared information and advice about different problem management strategies. There were two subthemes: using the Internet for due diligence and using the Internet to get good cancer care.

List members had already enacted one problem management strategy by joining an ACOR list and encouraged each other take further action against the cancer threat. Survivors’ messages provided information and advice about effectively using Internet resources to obtain and manage cancer care. Senders clearly viewed the Internet as an essential tool for cancer-related problem management.

Information messages from survivors supported listmates in using the Internet for due diligence by providing specific content of interest to individuals with a similar cancer diagnosis. Senders posted hyperlinks to websites they thought salient for listmates. The most frequently mentioned websites included those that provided definitions of diagnostic terms, descriptions of treatment protocols, and information on treatment side effects. In some cases, websites were sponsored by the National Cancer Institute (NCI) or other government agencies. Members also frequently recommended other nongovernmental sites that offered information about clinical trials or cancer-related research projects, links to published reports of research findings, and facilities offering specific types of cancer treatment. In four lists, survivors posted messages in which they recommended online publications or books they believed were reliable and helpful sources of information. When members recommended books, they typically included links to online retail sites offering the book.

The second subtheme, using the Internet to get good care and social support, was found in four lists. Typically, these survivor messages shared information about how to use the Internet to get the best possible care for particular cancers. Survivors also directed listmates to websites that contained information about providers from whom senders had received good care. In addition, survivors helped listmates find sources of social support, including other cancer support lists. At the same time, senders reinforced listmates’ participation in ACOR mailing lists by noting how to use listmates as resources. Senders often said they valued ACOR mailing lists because they provided access to more experienced members who could help them interpret test results and correct misinterpretations about published cancer research studies. In addition, senders noted how ACOR listmates provided practical information about the daily struggles of coping with cancer, coping at different stages, and the realities of long-term survivorship, all of which was a valuable supplement to the advice received during all too brief contacts with health care providers.

#### Theme 1.4: Cancer Recurrence

If survivors are fortunate, their treatments result in cures or very long periods of remission. However, being declared disease free does not mean these individuals can return to their precancer lives, so both fear of relapse and coping with relapse were important concerns. The major theme of cancer recurrence was discussed in six lists and included two subthemes: managing risk of recurrence and coping with relapses.

In five lists, survivors discussed problem-focused coping with the ambiguity of being found NED (no evidence of disease) or NERD (or no evidence of recurring disease). They admitted that they remained anxious about the possibility of recurrence, and reminded their listmates that, even after being declared disease free, they must remain vigilant for early signs and symptoms of recurrence. They also advised using due diligence at this stage by being proactive and staying informed about new treatment options, probability of cure and recurrence, and treatment side effects and after effects. They encouraged listmates to take steps to minimize the risk of recurrences. For example, senders recommended lifestyle changes, such as dietary changes, to reduce the likelihood of recurrence of colon polyps. In some lists, senders wrote about the importance of regular self-monitoring, noting that occurrence of a primary cancer increases the risk of subsequent ones, sometimes in different forms. They shared information about procedures for monitoring and discussed how to cope with the uncertainty of fluctuating test results and how they could use test findings to decide whether to seek treatment to prevent a recurrence. In one list, a member discussed the difficulties inherent in using statistics to assess an individual’s prognosis and the importance of regular monitoring:

Since [cancer type] patients come in all shapes and sizes, some with very aggressive disease on diagnosis, others smoldering for many years; it's impossible to have a level playing field.... My internist FIRST noticed a slightly elevated protein level when I became his patient…. Prior to that, no tests were ever taken that would break down this figure. It wasn't until [date removed] that this doc felt the protein level needed to be checked by a hem/onc, who decided that I was “smoldering.” I finally started treatment in [date removed] and had a transplant in [date removed]...one of the first combined stem cell and bone marrow transplants. Though I have relapsed twice, I'm still kicking.

In three lists, survivors reported how they coped with relapses. Although all felt emotionally devastated when informed about their recurrence, senders agreed the only way to cope was to keep in mind that they had to deal as well as possible, which included getting all the information they could. One member offered an example of how staying informed about treatment options enabled her to help her children adjust to the reality and implications of her relapse. Other survivors, such as the participant quoted earlier, provided information to sustain hope when confronted with relapses, reminding listmates that—despite multiple relapses—they were “still kicking.”

### Emotional Support Themes

#### Defining Emotional Support

Compared to the detailed technical discussions of cancer treatments, the emotional supportive text of survivors’ messages was often relatively brief and typically occurred at the end of messages. To categorize the emotional aspects of support survivors offered to others, we adapted Cutrona and Suhr’s [47] typology of emotional support (Tables 4 and 5).

**Table 4:** Categories of emotional support (adapted from [47])

**Type of** **Emotional Support**	**Description**
Emotional coping strategies	Suggestions for managing painful emotions (eg, anxiety, anger, fear, and sadness)
Empathy	Acknowledging identification with others’ emotional reactions and feelings including painful and pleasurable feelings; validating the appropriateness of another’s reactions to stressful circumstances
Encouragement	Support in persistence in facing challenges; expressing hope that situations will improve
Prayers	Offering spiritual support through prayers or blessings to others in distress
Esteem support	Appreciation for the value of an individual and his or her accomplishments

**Table 5:** Emotional support themes

**Theme Number**	**Theme**	**Number** **of Lists**
2.1	Encouragement	10
2.2	Empathy / emotional validation	10
2.3	Emotional coping strategies	10
2.4	Esteem support	9
2.5	Prayers	7
2.6	Solidarity	7

#### Rates of Emotionally Supportive Communication Across Groups

**Figure 3:**
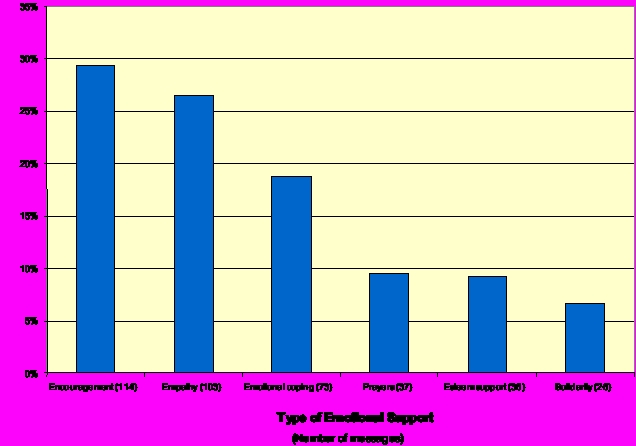
Types of emotional support offered by survivors (N = 389 messages)

As Table 2 shows, the 10 lists can be roughly divided into two groups along the level of emotionally supportive communication. Seven groups (L-T survivors, CLL, LM sarcoma, kidney, colon, and ovarian) were more supportive, with 19% to 30% of messages containing emotionally supportive comments. Emotionally supportive messages in the three less supportive lists (myeloma, lung NSCLC, and prostate) ranged from 10% to 13% of all messages. In addition, types of support varied across lists. Encouraging and empathic comments, as well as suggestions for emotional coping strategies occurred, to some degree, in all groups. In addition to encouragement and coping strategies, members in a majority of the groups offered several other kinds of emotional support (Figure 3). However, compared to encouragement and emotional coping strategies, the other types of emotional support occurred much less frequently.

#### Theme 2.1: Encouragement

As befits support groups, the most common type of emotional support was encouragement; about a quarter of messages included some encouraging notes. Frequently, these were simple exhortations at the end of messages, such as “Hang in there” or, for those embarking on a course of treatment, “Hope everything goes well for you!”

Others offered warm wishes for continued good recovery and “continued shrinkage” of tumors. Many messages encouraged the intended recipients to take a more activist attitude in the face of difficult circumstances. In all groups, members encouraged each other to persevere and to persist in staying vigilant for recurrences, seeking appropriate treatments, and overcoming obstacles to getting Social Security support benefits. A fourth type of encouragement served to sustain hope that cancer is treatable, that the intended recipients are “in good hands” at their respective treatment facilities, and that feeling better and continued good quality of life are achievable goals.

#### Theme 2.2: Empathy and Emotional Validation

Empathy is the ability to understand and identify with another person’s feelings in both happy and sad occasions and to respond compassionately to another’s distress [48]. We categorized empathic statements as those in which survivors wrote messages such as “The things you write are the same we’ve been through” and “We know how it feels to fight this beast.” We included comments that validated other members’ emotional reactions to the experiences as cancer survivors, such as “Like you, I am always scared before getting test reports” and “You can be pissed off and let off steam.” Survivors also expressed sympathy for each others’ disappointments, most often related to reports of recurrences or metastases. Happily, survivors also wrote when they had good news, such as good test results, announcements that they had survived yet another year, weddings, births, or other positive events. Listmates responded to these messages with expressions of pleasure and sincere congratulations, often mentioning how important hearing good news was to sustaining their hope for the future.

The most solemn types of emotional support were expressions of sympathy and condolences offered to caregivers and their families when loved ones died. These occurred primarily in three lists (LM sarcoma, L-T survivors, and ovarian). In instances in which the deceased had been an active member of the list, listmates sometimes responded with moving tributes: “[name deleted] was such an inspiration to us, always sending articulate and intelligent posts. We learned so much from our association with [name deleted] and the knowledge he shared.”

#### Theme 2.3: Emotional Coping Strategies

ACOR list members provided models of successful emotional coping by offering descriptions of attitudes and cognitions that helped them in their journey. Many messages encouraged self-acceptance, emphasizing that each person’s experience is unique and there are “no right or wrong ways to do it.” Similar advice on self-acceptance was offered for coping with treatment side effects and after effects: “Accept the new you” and “I enjoyed having my straight hair come back curly after treatment.”

Members frequently wrote about how their life view was transformed by the cancer experience, moving from dread to deep appreciation for the preciousness of life and the people they loved. One member described how keeping a journal of her cancer journey helped her find new meaning in life: “Strange that we have to suffer to find ourselves.” Members consistently stressed the importance of maintaining a positive attitude, and many wrote about the importance of experiencing life more fully. Members reminded each other to not only plan for the future, but also live life for today and not defer pleasure, such as long-planned vacations.

Members also described positive coping by using social comparisons. They could tolerate treatment side effects if it kept “the beast at bay,” bolstered by the prospect of feeling better. Listmates offered examples of people who had lived long lives and accomplished much even when living with cancer.

Members also offered practical advice. One suggested that listening to music could reduce feelings of claustrophobia while being scanned. Others recommended managing anxiety by recalling images of favorite peaceful places or seeing a psychiatrist to manage guilt and the anxiety of “living in limbo after chemo.”

#### Theme 2.4: Esteem Support

We defined esteem support as verbal expressions that specifically built a sense of self-worth, value, and competence. Survivors nurtured their listmates’ self-esteem by explicitly acknowledging how frequently their messages served as sources of expert information, strength, and inspiration, as well as models of courage and coping. Various members commented how messages had helped them cope at different stages of their illnesses: when newly diagnosed, when confronting a new course of treatment, and at the terminal stage (for example, “How you inspire us with your courage and wisdom as you face what we all must.”). In addition, members expressed appreciation for those who continued to participate in the lists and provide support for others even after they had been declared NED (no evidence of disease). Survivors also commented appreciatively about listmates’ individual characteristics, such as courage, assertiveness, humor, and a positive attitude, thus reinforcing strengths that would help them cope with their cancer. Not surprisingly, members also valued good writing and members who could “verbalize what so many of us feel.”

#### Theme 2.5: Prayers

Survivors who offered help were relatively secular in their support. Across all groups, prayers and blessings were relatively rare (Table 5), and we did not find any in three of the lists (kidney, lung NSCLC, and prostate). In contrast, almost half of survivor messages sent in the ovarian list contained a reference to prayer. The type of spiritual support offered in these messages tended to be brief, conventional (although certainly sincere), and included phrases such as “God bless you” or “You and your family are in my thoughts and prayers” as part of the message closing. In the section on listowner facilitation roles below, we discuss factors that may have deterred members from expressing more religiosity.

#### Theme 2.6: Solidarity

We found the final type of emotional support, solidarity, scattered across survivors’ messages in seven of the lists. This type of support included general expressions of solidarity so members would not feel alone in their treatment tribulations. As with prayers, half the messages containing expressions of solidarity were sent by members of the ovarian list. Members wrote expressing interest in learning how others in the list were faring with treatment, but most often these expressions occurred as simple statements of support such as “I will be thinking of you” or “I wish the best for you.”

### Listowner Facilitation Roles

Across all mailing lists, discussions appeared to be very task focused. Neither survivors nor caregivers shared much information about their families or jobs or other aspects of life not directly affected by cancer. This raised the question of how members learn what is appropriate for discussion. We queried ACOR listowners by email about this issue, and they informed us that they worked both online and offline to facilitate discussions (Table 6). ACOR mailing lists function as a loose federation; listowner teams on each list have the authority and flexibility to shape their respective lists according to their shared personal visions and understanding of list needs. Listowners also have a closed-membership list of their own called Oncolist. Participation in Oncolist provides listowners with access to the knowledge and experiences of other listowners specific to facilitation of cancer-related mailing lists.

Although the content of each list varies substantially, ACOR provides a framework for some standardization. For example, all new subscribers to ACOR lists receive an automated welcome letter from Gilles Frydman, ACOR's founder. In addition, each list sends out an automated welcoming message that describes the group’s intended audience as well as its purpose, but it generally does not specify topics that are unacceptable to the list community. In some instances, listowners have added brief, automated signature line reminders about list netiquette, such as this posting from the lung NSCLC list: “Support is our goal. Please, no: Commercial, religious, political, rude, or off-topic messages; no unapproved questionnaires.”

ACOR listowners who participated in our study reported that they preferred not to actively direct discussion content because it could be disruptive and provoke flaming.

**Table 6:** Listowner facilitation roles

**Role**	**Description**
**Modeling appropriate Behavior**	We try to model appropriate behavior by offering praise and thanks off-list to those members who have been helpful to others. While we don't “coordinate” these efforts, we do copy each other on our private messages to members so that there is less chance of overlap/duplication. I think we also “model” appropriate list behavior with our own posts to the list (by, for example, staying on topic, trimming prior text, and the like). (ES, personal communication, November 10, 2005)
**Keeping discussion focused on cancer-related topics**	We also try to limit humor and cute stories as much as possible, because while some people like them, there are other venues for that and they take up a lot of space. Anyhow, they irritate all three listowners and it isn’t a democracy ;-). (DB, personal communication, November 11, 2005)
**Enforcing group norms**	I do occasionally send out private messages to individuals that have posted inappropriate material…. Usual causes for personal messages are posts containing personal attacks of individuals, expressions of inflammatory political opinion, commercial content, etc. Haven’t seen any controversial religious stuff in quite a while. I do feel that when the discussion establishes at a level of higher quality that it tends to sustain itself and little intervention is need on my part. If an eccentric individual begins posting inappropriate or provocative material, this tends to invite response, and dialog can deteriorate. (CC, personal communication, October 28, 2004)

List members often remind each other about rules, reducing the need for active interventions by listowners. Mailing lists that are part of a portal have an advantage when they encounter a member who persistently goes off topic, because they can easily redirect that member, encouraging him or her to join a list elsewhere on the portal where members' interests would be more convergent. For example, if a member frequently posts religious messages beyond conventional requests such as “Please pray for me,” listowners may urge the member to join another ACOR list, such as the Cancer Survivors Christian Online Support Group, which specializes in Christianity and cancer. Similarly, members seeking support for use of alternative cancer treatments have the option of joining the CAM-ONC list, which provides a forum to discuss how to integrate complementary and alternative cancer care with conventional approaches.

## Discussion

We found consistent patterns across the lists in participants’ behaviors, focal topics of discussion, and in the types of support they offered each other. In this section, we focus on: (1) factors related to supportive discussion content across lists aimed at helping list members cope with their diseases and treatment effects, (2) methodological issues related to project sample size and participant characteristics, and (3) new automated methods to help researchers analyze the large quantities of data generated by online communities.

### ACOR Mailing Lists as Sources of Support

#### Implicit Support

An important component of our investigation was to examine the functions of implicitly supportive messages and the role they play in overall support offered through lists. Preece argued that personal narratives and case histories are examples of empathic communication [49]. Unsolicited case histories can strengthen the group’s solidarity and reduce individual members’ sense of isolation by allowing other members to know how others are weathering their cancer-related challenges. Further, when members send messages that include URLs of resources, they remind the list of resources that are accessible via the Internet, making it easier for their listmates to engage in coping using problem management strategies.

#### Dominance of Offers Over Requests

We were surprised to find large and consistent differences in the proportion of messages offering support compared to those explicitly requesting support. However, based on the few studies that explicitly described list-based offers and requests for support, this behavior may be the norm rather than the exception [21,50,51]. Although researchers have presented their data in ways that make it impossible to make direct comparisons, there are conceptual similarities. In studies examining support-seeking behavior, the percentage of messages containing offers of informational support ranged, at the lower end, between 37% (problem drinkers) [21] and 40% (primary biliary cirrhosis) [50], and at the higher end, between 80% (colon cancer) [52] and 85% (diabetes) [51]. Our findings are consistent with those of earlier researchers who found offers of emotional support were less common, ranging from nearly 29% (problem drinkers and diabetes) [21,51] to 48% (colon cancer) [52]. Similarly, in these same studies, there were proportionately more requests for information, ranging from 15% (problem drinkers) [21] to 23% (colon cancer) [52]. Messages containing requests for emotional support were rare in all these studies, ranging from 2% to 3% of all messages. Populations and methodologies in these studies were so disparate as to make it difficult to draw strong conclusions about these differences.

We cannot determine the reason for these patterns. One possibility is that many ACOR list members do not have to ask for help because their offline resources for support are adequate. Within ACOR they can get answers on many topics or find role models for coping simply by reviewing list archives or lurking long enough to read exchanges of messages between others who are facing similar challenges [53]. Also, email communication is asynchronous and allows senders to remain invisible and anonymous. These restricted channel characteristics promote idealized views of senders [46]. Further, senders can choose their words and manage how they present themselves to the lists. Given the choice, it is likely that most people would choose to present themselves in a positive light. However, many of these people were under extreme stress, and that tends to reduce the likelihood of positive framing. Finally, being able to offer help is a more empowered position than being a supplicant. List members who have more personal experience with cancer and have learned a lot about cancer from participating on the list can find rewarding roles as “elders,” sharing both what they have learned and how they have coped [54].

### Factors Affecting the Range of Discussion Topics

#### Information and Advice About Cancer Treatments

The combined findings from prior research on ways people use the Internet, the stated missions of mailing lists, as well as new ACOR subscribers’ self-reports about what they were seeking by subscribing to the lists led us to expect that the dominant themes would focus on cancer-specific treatments. This hypothesis was borne out by our analyses. However, ACOR members presented this information in ways that suggest they were inventing their own form of evidence-based medicine. Members shared information and advice about clinical trials, their own experiences with side effects and after effects of specific treatments, and the practical points of managing different treatment [55]. With this information, list members felt better prepared to make informed choices, to become active members of their own care teams, and to negotiate with doctors and medical teams about which treatments to pursue. Similarly, by being better informed they could cope more effectively with the effects of treatment as well as with recurrence when treatment failed. Nevertheless, we did not have data by which to evaluate the quality of decisions participants made.

Typically, discussions about qualitative research findings remind readers that findings are based on convenience samples and are not generalizable to other situations or populations. However, we believe we can make stronger claims about the external validity of our conclusions. Our results are supported by the results of our quantitative Health eCommunities surveys of new ACOR subscribers, which found that they were most interested in informational support. Our results are also consistent with findings from several other studies that included analyses of list member communication across diverse health support lists whose discussions focused strongly on treatment-related issues [21,51,54,56-58].

#### Communicating With Health Care Providers and Finding the Best Treatment

People are unlikely to subscribe to illness-support lists unless they—or people they care about—have an illness. List members’ messages were somewhat more likely to address issues about what to talk about with their doctors than about finding treatments. Many list members had already been diagnosed and were embarked on some kind of cancer treatment journey. Members reported examples of both good and bad relationships with their treatment providers. In general, discussions about finding treatment providers were more often about doctors and institutions offering the *best* treatment.

In the majority of lists, members focused on what they should discuss with their doctors. In five lists, some members expressed concerns about recommendations their listmates received from physicians and advocated seeking second opinions. In the literature, there have been reports on the reactions of providers whose patients bring information obtained from the Internet to the clinical encounter [59]. However, in our data, there was little reporting about list members bringing information and suggestions from mailing lists to their doctors or about how their doctors responded. It might be helpful to develop materials for listowners with suggestions about how to communicate optimally with physicians. Such information could be shared among lists.

#### Virtual Community Support for Active Coping

In our conceptual model of stress and coping (see Figure 1), list members’ levels of self-efficacy and their information-seeking styles (illness appraisals) play a role in their coping strategies. For message analysis, we lacked baseline assessments of these attributes. We hypothesized that participation in the lists and social support provided by lists would impact coping efforts. Because we did not have pre- and post-measures of individual illness appraisals or coping styles for these quantitative analyses, we could not determine the extent to which participation in lists caused members to change their coping behaviors. However, these data show that when cancer-affected people join ACOR lists, they are consistently encouraged to engage in active coping behaviors (eg, information seeking, asking questions of health professionals). Members encouraged each other to maintain this “activated” stance at each stage of their treatment, regardless of whether their cancer was active or in remission. Moreover, list members were provided models of how they could use the Internet for due diligence in learning about their particular form of cancer, the most effective treatment options, and the institutions or providers from which they could obtain good care.

#### Emotional Support

List members offered each other encouragement, empathy, and shared emotional coping strategies. Given how frightening the prospect of cancer and cancer treatments can be, it is somewhat surprising that comparatively few of the messages contained explicit, emotionally supportive content (7% to 18% of messages). In addition, although messages were relatively restrained in emotionality, we found several kinds of emotionally supportive content in the messages, possibly increasing their impact.

Overall, survivors’ emotionally supportive messages reinforced list values for active coping strategies. Survivors often couched their messages in optimistic, activist, or even militant terms (eg, “Never give up the fight!”)*.* Moreover, messages offered esteem support for active members who modeled active coping. Survivors who offered support also recognized that there were situations in which active coping strategies were not appropriate. They acknowledged that coping strategies had to fit individual situations, modeling cognitive reframing strategies to cope with existential situations rife with uncertainty or many uncontrollable factors. ACOR members tried to provide help by suggesting coping strategies appropriately tailored to their listmates’ situations.

### Methodological Issues

#### Ethical Issues in Qualitative Research on ESGs

Among researchers studying online groups, one of the ongoing debates is whether mailing lists and other online groups are “public spaces or private rooms” [39,60]. This issue has major ethical implications for how qualitative research is conducted in these groups. Eysenbach and Till [60] have suggested that when some form of registration is required to gain access to the list, then most subscribers are likely to regard the group as a “private place.” They also note, however, that members may view groups with a larger number of subscribers (> 100) as less private than those with fewer subscribers. Qualitative researchers who want to use email message texts from existing groups are confronted with the challenge of obtaining informed consent from members who did not join the lists to participate in research. While researchers may be able to obtain listowner permission to use messages, this strategy does not allow individual list members to give their consent. Once the data are analyzed, if verbatim quotes from members’ messages are used in publication, then the researchers are obligated to reduce the risk of unwanted exposure by removing all identifying information from message texts and to obtain the senders’ permission to use them.

In this study, we made systematic efforts to protect participants’ rights at each stage of the research. We only recruited larger lists (> 200 subscribers). Although new subscribers to ACOR lists have to register, the ACOR website informs all newcomers that that ACOR archives are publicly accessible. As a result, participants in this study were more likely to view their lists and list archives as public rather than private spaces, and less likely to resent having their messages used in research.

With regard to consent, we had listowners’ permission to use messages from their archives and we notified subscribers about the project’s online fact sheet. The scale of the study prevented us from obtaining individual consent from all subscribers. In any case, members could request to have their messages removed from the analysis, but none did.

Although we did not promise to request permission to use quotes, the sheer scale of the project protected participants’ privacy. The goal of the study was to identify commonalities in the provision of support across lists so the data were summarized extensively. Ultimately, we used very few direct quotes and most of those were only brief phrases. In the few instances in which we present more extended quotes, we removed information linking the quote to cancer types or specific lists.

#### Sample Size

Posts were highly variable in timing and frequency. It was an empirical question whether systematic sampling would capture this variability. As it turned out, we underestimated activity levels within lists (see Figure 2). On a gross level, comparing mean activity levels between the sample and full data sets, sample activity levels were higher in 7 of 10 lists. In general, these average differences ranged from one to two messages overall per month, with the one exception of the myeloma list, where the difference was approximately five messages. The ovarian list was the only list in which the sample averages were somewhat lower than those of the full list dataset.

We do not know the reasons for these differences between the sample and full data sets. One possible explanation is that, when more active members went online, they initiated more new messages *and* posted replies to other frequent senders during relatively short time periods. With our systematic sampling method of extracting messages from chronologically sorted sample messages, we may have tapped into a somewhat greater proportion of those active members’ posts.

Differences in participation rates between groups may also have other implications for the way support is experienced by members in different lists. If the typical member only posts one or two messages a month, they may be less likely to use those messages to comment on or to reply to others’ messages. A consequence of infrequent reading of list email is that individual senders are less likely to see replies made to their own postings. In turn, listmates whose response fail to elicit follow-up comments or answers to a posed question may come to regard nonresponsiveness as the norm for the larger mailing list community. As a result, members may experience the list as less responsive to individual needs.

#### Participant Characteristics

Group composition was another source of variability across lists. Similar to many other Internet-based organizations, ACOR does not collect background information on its subscribers. Thus, we did not know whether subscribers were survivors or cancer-affected others. We could only deduce their identity as survivors or caregivers from what they wrote in messages. We believe we were successful in doing so in most cases. The median number of messages for which we could identify senders’ roles was 87%, but this proportion varied across lists. We were able to identify the most sender roles in the lung NSCLC list (92% of messages) and the fewest in the CLL list (80% of messages).

We found, as expected, that the majority of messages were from cancer survivors. However, there was also considerable variability in the proportion of messages sent by those whom we identified as family caregivers, ranging from a low of 5% in the ovarian list to nearly half (48%) in the esophageal list.

Lists with the highest proportion of caregiver messages were also the ones serving survivors with the most potentially deadly cancers (eg, esophageal). This finding is consistent with other research showing that Internet users who were support seekers were more likely to be caregivers than people suffering from health problems [6]. Possibly, these situations occurred when the survivors who originally subscribed to the list became too ill to post messages, so their caregivers took over. Also, as survivors get sicker, their caregivers may need more support and so post more frequently to their respective lists, thereby increasing the likelihood they would be included in our sample. If caregivers feel isolated due to demands of care giving, lists may serve an especially important role. As with other structural variations in ACOR lists, our explanations for these differences are speculative.

Although introductory information in list welcoming messages specifically mentions that health care providers are encouraged to participate, we found little evidence that they did. For the most part, those identified as health care providers were also cancer survivors. In a few cases, information in message texts or footers suggested that the health care provider sending the message had drawn on both personal and professional experience to become a cancer patient advocate or was involved in producing educational materials for patients.

Prior research indicates that many health professionals are concerned that patient-provider relationships will be affected when survivors bring information found on the Internet to discuss during appointments [59,61]. If health professionals participate in health support mailing lists as participant observers, they may become better informed about how lists function and may be more effective in helping survivors maximize the benefits of list participation. Surveys of physicians [61] and new ACOR members [30] showed that many survivors are taking health information they find on the Internet to discuss with their physicians. Although we found reports of physicians participating in online clinical discussion groups [52], we were unable to find studies of physician participation in online illness support groups with survivors and caregivers. Across all lists in our study, the median number of messages from identifiable health care providers was 1%. In five groups, no messages had senders identifiable as health care professionals. The prostate list had the largest proportion of messages sent by health care providers (4%). Further research is needed to identify barriers to participation by health care providers as well as ways to increase the motivation of health professionals to become active in online support groups. Potential barriers include health care providers’ overloaded practice schedules, lack of incentives to seek information about online support resources, and potential liability issues associated with offering medical advice or opinions online.

#### External Validity

What does external validity mean in the case of naturally forming peer to-peer groups on the Internet, such as those in ACOR? Here we define it in terms of what new list members can realistically expect to find when they join a group. Early research suggests the pattern of predominance of offers of support over requests that we found among participants on ACOR lists is similar to those in other groups [21,50,51]. However, the pattern of messages containing much more informational support than emotional support probably reflects the highly technical nature of cancer treatments. Further research is needed to determine whether online groups for people living with chronic health and mental health conditions focus less on treatment technologies and more on emotional support for coping.

ACOR’s organizational structure is another factor that could limit whether such health-related ecommunities can be replicated. ACOR’s mailing list for listowners (Oncolist) appears to play a key role in developing and sustaining organizational capacity*.* Oncolist serves as a forum for listowners’ discussions on the technical management of ACOR’s Listserv software, virtual organizational development, and discussion facilitation as they relate to cancer-affected people. Listowners report that they value the information and support they receive from the list and read messages from the list regularly. Oncolist supports the long-term viability of all ACOR mailing lists by providing ongoing training for new listowners and encouragement for experienced ones. Through the list, listowners are also alerted when a listowner’s deteriorating health or decision to step down precipitates a staffing problem on another listowner team. Some listowners report that they participate on multiple ACOR listowner teams, sharing their experience and reducing other listowners’ workloads and the risk that a list will dissolve due to loss of leadership.

Other major Internet providers that host online communities have already recognized the need for such groups. Both Yahoo Groups and Google Groups host lists called “EL-M” (ElectronicMailingList-Manager) for listowners. However, these EL-M lists are open to an extremely heterogeneous collection of lists on widely divergent topics. Apart from our own preliminary unpublished studies, we could find no reports of research that tracked the organizational development of health eCommunities over time. In this context, it remains to be seen whether analogs to Oncolist will develop on Yahoo, Google, and elsewhere on the Internet to address the specialized needs of listowners who oversee ESGs for related health conditions.

### Limitations

Email communication on mailing lists is fragmented. List members vary widely in how often they check their email and how often they post to lists. In lists with high volume, members can easily miss messages sent in reply to their posts. By systematically sampling the sequence of messages within list archives, we further fragmented the sequence and context of member correspondence. Although we expected that this sampling technique would, on balance, tap into correspondence of the most active members and identify the most common themes, we did not have the resources to confirm our interpretations with the full data set. Even though we sampled messages over a five-month period, our findings are summative. Discontinuities caused by our sampling techniques also prevented us from tracking within-group thematic changes over time. These situations are prime examples of the critical need for automated language analysis tools to examine individual sender behaviors within the rich context of online discussions as they occur over time.

Our analyses of participant characteristics revealed that caregivers were important participants in ACOR communities. It is beyond the scope of this article to present the perspectives of caregivers, as givers and recipients of support, but we will report on this topic in the future.

Finally, the mass of complex textual data produced by this study pushed the limits of conventional qualitative data analysis methods. To compare 10 lists in a single report, we necessarily omitted finer-grained descriptions of phenomena within individual lists. Our systematic sampling strategy constituted a trade-off. It supported this kind of macro analysis but made it impossible to ascertain whether messages that we used as the basis of identifying themes really represented dominant threads within the groups or were artifacts of the sampling process. The scale of the study also limited the extent to which we could let list members “speak” for themselves through their email texts. Given the space allowed in most journal articles and the number of themes identified, we were limited to summarizing texts and offering exemplars. As with the demand for new analytic technologies, large-scale studies of Internet communities may also require new formats to document the discoveries we make about them.

This project used procedures to de-identify messages and perform systematic sampling to obtain data for each month in a timely manner. However, even using a sophisticated qualitative data analysis program like Atlas.ti to analyze messages, it took months to complete analyses because of the volume of messages and the number of coding categories we used. This slow process of data analysis contrasts sharply with the speed of the online communications that produced data. If research is going to accommodate the scale and scope of burgeoning eHealth support resources like mailing lists, finding ways to automate analytic processes will be essential. Information and social scientists, such as Pennebaker [62], Arguello [63], Kraut [personal communication, January 15, 2006], and Seale [64], have been working independently to develop efficient text data mining procedures that will work with semi-structured textual data, such as email messages [65,66]. As new technologies emerge, researchers will be able to study mailing list member interactions more comprehensively, over longer periods, and using a variety of methods. Thus, these innovative tools are critical to our capacity for rapidly gaining insights into the processes and discussion content of these groups. Ideally, in the future, researchers will use mixed methods—qualitative methods to explore the depth and richness of qualitative data and quantitative methods to achieve the potential precision and generalizability of quantitative data.

### Conclusions

We examined the ways in which 10 cancer lists provide informational and emotional support. We found that, across all lists, cancer survivor participants were more likely to offer support than ask for it. Survivors communicated support primarily through offers of technical information and explicit advice about how to communicate with health care providers to get optimal care. Survivors offered support implicitly through accounts of their illness and treatment experiences. Explicit emotional support was less frequent than informational support and was often embedded in message texts. Emotional support often encouraged active coping, providing opportunities for cancer survivors to play empowered “helper” roles that reinforce self-esteem. Internet health support seekers report they are looking for both information and emotional support. This study offers strong evidence that they can and do find the support they need—and can, potentially, benefit from opportunities to play empowering roles in their online communities.

## Abbreviations

ACOR: Association of Cancer Online Resources
CLL: chronic lymphocytic leukemia
ESG: electronic support group
UNC: University of North Carolina at Chapel Hill
